# Biogenesis of protein bodies during vicilin accumulation in *Medicago truncatula* immature seeds

**DOI:** 10.1186/1756-0500-5-409

**Published:** 2012-08-04

**Authors:** Mona Abirached-Darmency, Fabrice Dessaint, Emilie Benlicha, Charles Schneider

**Affiliations:** 1 INRA, UMR1347 Agroécologie, BP 86510, F-21000, Dijon, France

## Abstract

**Background:**

Grain legumes play a worldwide role as a source of plant proteins for feed and food. In the model legume *Medicago truncatula*, the organisation of protein storage vacuoles (PSV) in maturing seeds remains unknown.

**Findings:**

The sub-cellular events accompanying the accumulation of vicilin (globulin7S) were analysed during seed mid-maturation. Immuno-detection of vicilin in light microscopy, allowed a semi-quantitative assessment of the protein body complement. The identified populations of vicilin-containing protein bodies are distinguished by their number and size which allowed to propose a model of their biogenesis. Two distributions were detected, enabling a separation of their processing at early and mid maturation stages. The largest protein bodies, at 16 and 20 days after pollination (DAP), were formed by the fusion of small bodies. They have probably attained their final size and correspond to mature vicilin aggregations. Electron microscopic observations revealed the association of the dense protein bodies with rough endoplasmic reticulum. The presence of a ribosome layer surrounding protein bodies, would support an endoplasmic reticulum–vacuole trafficking pathway.

**Conclusions:**

The stastistic analysis may be useful for screening mutations of candidate genes governing protein content. The definitive evidence for an ER-storage vacuole pathway corresponds to a challenge, for the storage of post-translationally unstable proteins. It was proposed for the accumulation of one class of storage protein, the vicilins. This alternative pathway is a matter of controversy in dicotyledonous seeds.

## Background

Grain legumes accumulate large quantities of seed storage proteins which are of worldwide importance as protein sources for feed and food. The mechanisms of transport and packaging into storage vacuoles, which have generated intense debate, require a complex cellular machinery. Plant vacuolar trafficking involves multiple vacuolar sorting determinants and mechanisms with the involvement of specific signals and receptors [1,2]. The processes of protein storage trafficking and protein storage vacuole formation have been studied in pea, beans and soybean, with indications of species differences concerning the mechanism of formation of protein storage vacuoles [3-6]. In *Arabidopsis* seed, different pathways may be implicated in storage protein accumulation [7], but, importantly, *Arabidopsis* lacks vicilin (7 S), one of the main legume storage protein types.

In *Medicago truncatula* seeds as for most other legumes, storage proteins are essentially synthesized and accumulated by embryo cells. At maturity, the cotyledons become the main storage tissue, the endosperm being degraded during seed development [8]. This model legume has benefited from large genetic and genomics programs [9,10]. The recent generation of tilling mutant populations, has reinforced the need for additional markers for grain phenotyping [11]. The timing of storage protein synthesis and accumulation has been analysed by in situ hybridization, transcriptomic, and proteomic approaches. The results showed that the two major seed storage proteins, vicilin (7 S) and legumin (11 S), are not synchronously synthesized [12,13]. Little information is available on the histological organization of the storage protein in the cotyledon cells.

In this cytological characterization of storage protein accumulation, we have focused on the ontogeny of the cytoplasmic organelles involved in the process at mid-maturation. The different populations of dense protein bodies which are assumed to mediate storage protein transport and accumulation have been identified and subject to quantitative analysis. Their kinetics of formation and quantification should help to elucidate the mechanism of storage protein accumulation in *M. truncatula* seeds. In addition they may be suitable markers for identifying alterations in storage protein accumulation due to genetic variation and environmental changes.

## Results

### Changes in embryo cell morphology during protein storage accumulation

**Figure 1:**
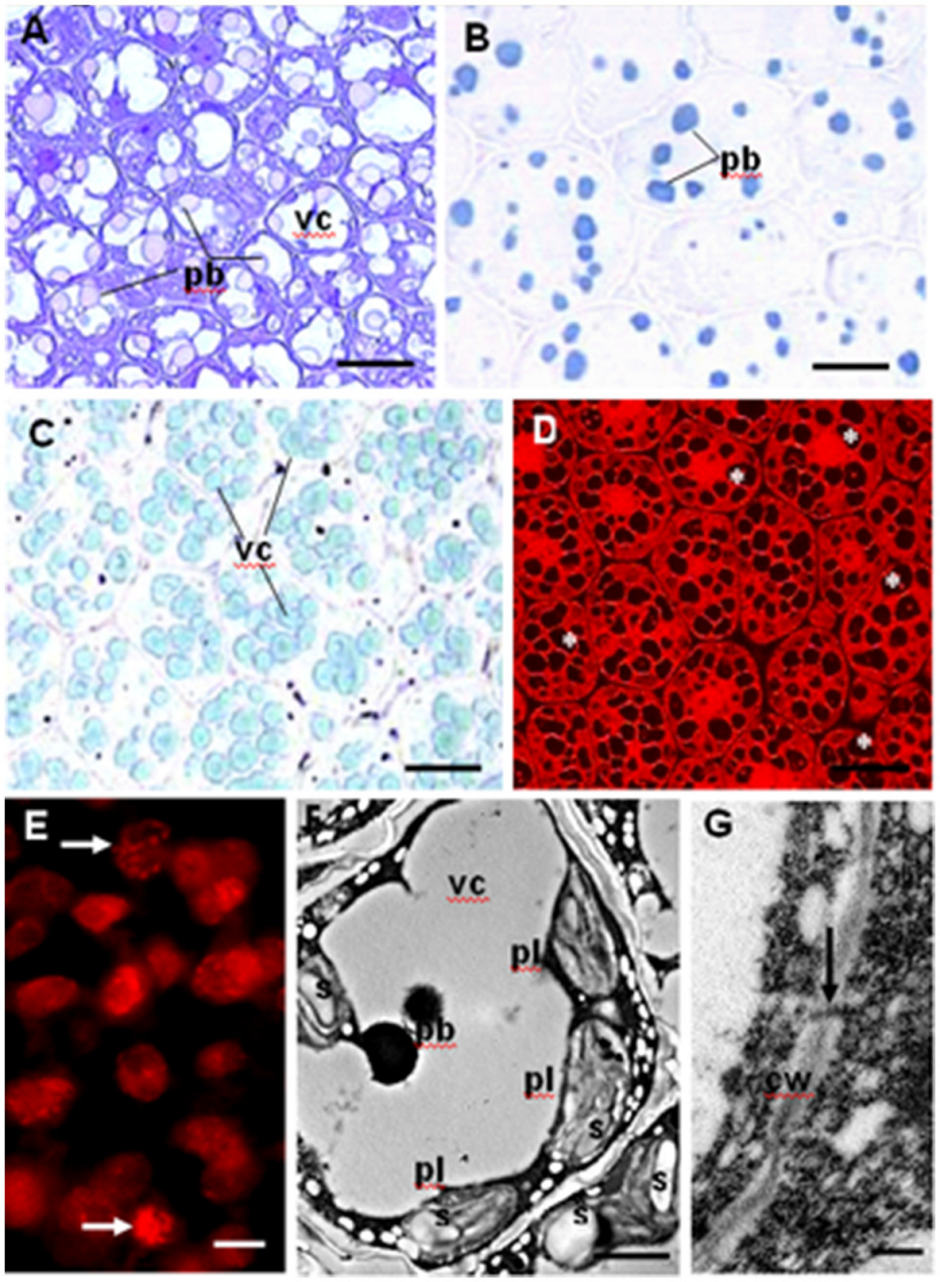
**Embryo cell micrographs in seed sections at early-mid maturation.** (A-D) Light microscopy detection and characterisation of protein bodies in seed sections. (**A**) The initiation of vacuole fragmentation is shown 16 DAP, storage bodies are pink due to the metachromatic Toluidine blue (TBO) staining. (**B**) The protein bodies (pb), revealed with Naphtol blue black protein staining, are more abundant at 20 DAP. (**C**-**D**) Illustrate the pattern of the vacuolar compartements (vc) at 20 DAP, with the fast green protein staining (**C**). They correspond to black holes (**D**) indicated by (*) using the fluorescent propidium iodide. (**E**) The mitotic figures observed in the abaxial region are indicated by arrows. (**F**-**G**) Electron micrographs illustrate the abundance of plastids (pl) and starch granules (s) in (**F**), the presence of plasmodesmata in the cell wall (cw) is shown in (**G**). The scale bar corresponds to 4 μm in **A)**, (**B)**; 8 μm in (**C)**, (**D)**; 7 μm in (**E)**; 1,5 μm in **F**; 0,4 μm in **G**.

At early maturation stage, the first dense protein bodies were identified in embryos 3 mm in length, and the initiation of a partition of the central vacuole, which became progressively a multiple vacuolar compartment, was observed (Figure 1A, B). By 20 DAP, in each embryo cell, the vacuolar architecture displayed 10 to 15 protein storage vacuoles in seed section (Figure 1C, D). A developmental gradient was evident within the embryo; the abaxial cell population was still dividing while the adaxial cell populations had initiated storage protein body accumulation (Figure 1E). Embryo cells were interconnected by plasmodesmata, they showed the characteristics of a high metabolic activity with the presence of abundant large plastids filled with starch and a massive proliferation of the endoplasmic reticulum (Figure 1F, G).

**Figure 2:**
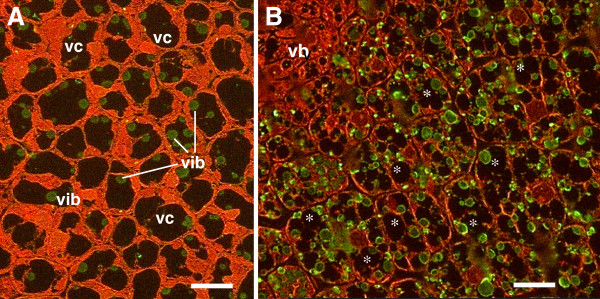
**Light microscopy immuno-detection : the anti-vicilin antibodies are visualised with Alexa 488 green fluorescenceand counterstained with the red fluorescent propidium iodide.** (**A**) Embryo cells 16 DAP showed green fluorescent vicilin bodies (vib) and the initiation of vacuole fragmentation. (**B**) Note an increase in the number of vicilin bodies and the presence of a multiple vacuolar compartment (*) at 20 DAP. vb, Vascular Bundle; vc, vacuolar compartment. The scale bar corresponds to 4 μm.

Immuno-localisation of vicilin, using anti-vicilin antibodies conjugated to the fluorescent Alexa 488 green tag, allowed us to follow the biogenesis of the vicilin-containing bodies during embryo maturation. In our experimental conditions protein bodies showed very faint autofluorescence and the anti-vicilin antibodies was preferentially localized at the periphery of the bodies which confirmed their specificity (Figure 2).

### Distribution of protein bodies at early and mid-maturation

**Figure 3:**
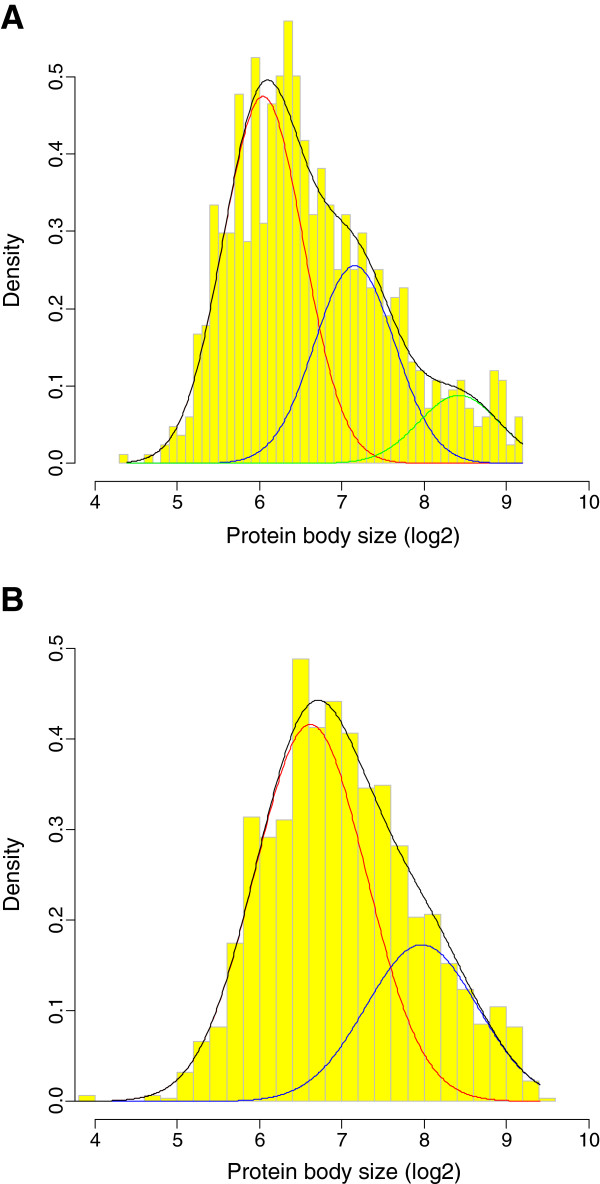
**Histograms for the log2-transformed protein body size data, with fitted density curves.** (**A**) At 16 DAP, the black curve is the fitted mixture density function, the red curve is the first component density function, the blue curve being the second component and the green one the third component. (**B**) At 20 DAP, the black curve is the fitted mixture density function, the red curve is the first component density function and the blue curve the second.

Image acquisition of vicilin-containing protein bodies allowed us, by applying statistical analysis on their number and size, to propose a model on their distribution and their kinetic formation during early and mid-seed maturation. At early maturation (16 DAP), as shown in Figure 3A and Table 1, the best model for characterizing the distribution of protein body size had 3 components of equal variance ($\sigma_{1}=\sigma_{2}=\sigma_{3}=[1]0:238$). The largest population (58%) corresponded to the small protein body size, it was clearly separated from the population with large size (10.8%). The third population (31.2%) displayed protein bodies of intermediate size. This distribution showed that the three protein body populations were readily distinguishable by differing size. On the logarithmic scale, the adjacent modes of the three groups differed by approximately one unit (in log_2_). The size difference between the three populations equals two units (in log_2_), which corresponds, in term of protein body size, to a factor four between the largest and the small protein bodies. This pattern suggests a two-step process in which large bodies are produced by fusion of the small ones.

**Table 1:** Parameter estimates for models in the comparison between the 16 DAP and 20 DAP populations

	**16 DAP**		**20 DAP**	
Component	${\hat{\mu }}_{k}$ (log_2_)	${\hat{p}}_{k}$	${\hat{\mu }}_{k}$ (log_2_)	${\hat{p}}_{k}$
1	6.04	58.0	6.62	70.7
2	7.16	31.2	7.97	29.3
3	8.42	10.8		

Please note that the size measurements are relative values. The ${\hat{\mu }}_{k}$ and ${\hat{p}}_{k}$ are the estimated location and proportion parameters for the *k*th component.

**Figure 4:**
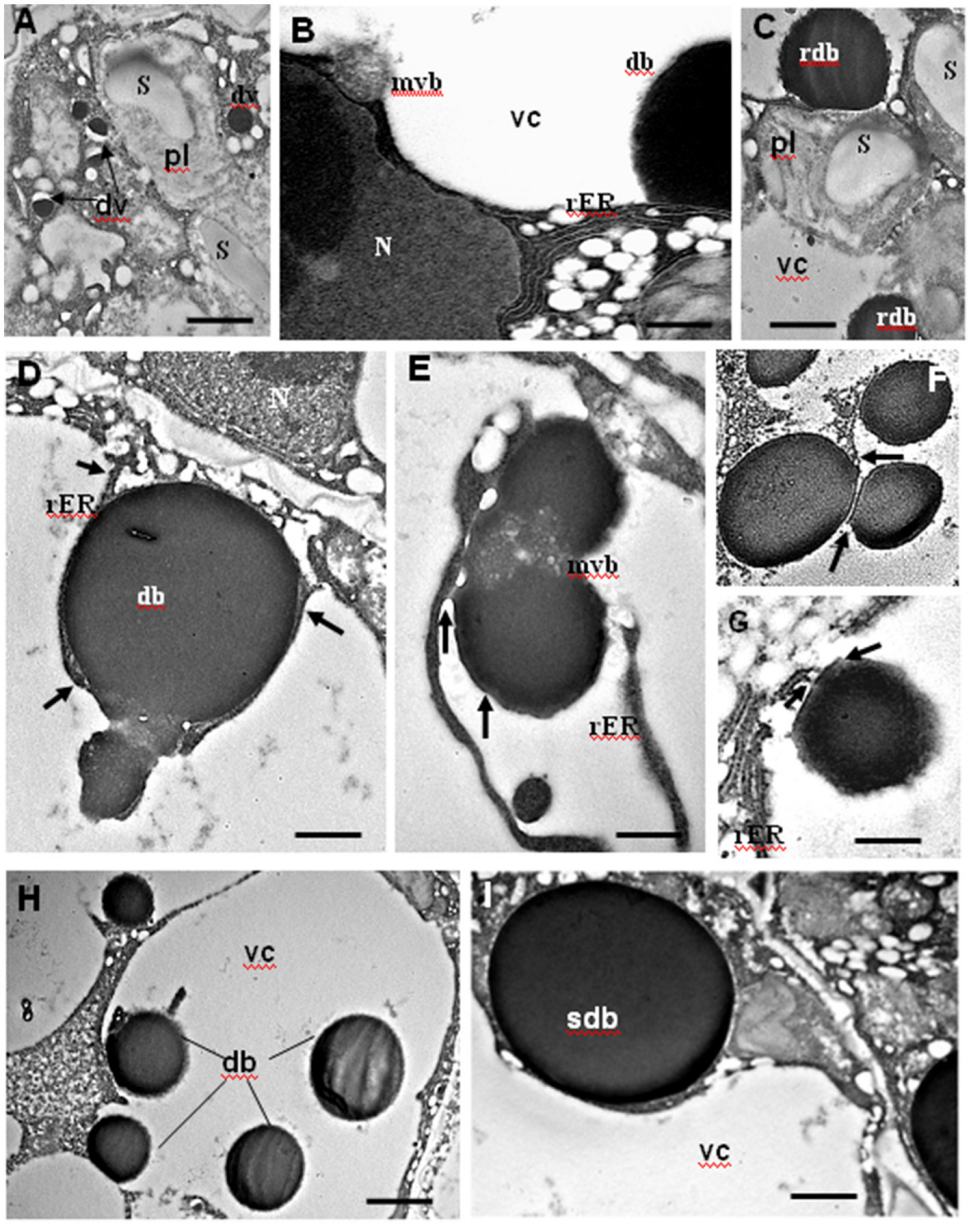
**Biogenesis of protein bodies at mid maturation as shown in Transmission Electron Microscopy.** The embryo cell ultrastructure revealed different Protein body cores. (**A**) Dense vesicles (dv) are shown in the cytoplasm (arrows). (**B**) The autophagy of a multivesicular Body (mvb) in the vacuolar compartment (vc) is shown. (**C**) Rough dense bodies (rdb) observed as an accumulation of precursor aggregates. (**D**, **E**) Illustrate the fusion of protein bodies through a multivesicular body (mvb) and with the association of the Rough Endoplasmic Reticulum (rER). The arrows in (**D**) point on the rER and in (**E**) on the ER derived bodies. (**F**, **G**) Remnants of the rER membrane associated with dense bodies were observed (arrows). (**H**) Embryo cell with different sizes dense bodies (db), which illustrate the proposed statistic model. (**I**) The large smooth dense body (sdb) observed in the cytoplasm, corresponds probably to its definitive size. N, Nucleus; db, dense body. The scale bar corresponds to 0,4 μm in **G**; 0,5 μm in **B**, **E**; 1 μm in **A**, **D**; 1,2 μm in **C**, I; 1,5 in **F**, **H**.

By 20 DAP, only two protein body size classes were maintained; the best model for characterizing the size distribution having two components of equal variance ($\sigma_{1}=\sigma_{2}=0.459$). At this stage, as for earlier stages, the predominant population (70.7%) corresponds to the smallest protein bodies which was clearly separated from the large sized population as shown in Figure 4B. However, the variance was higher than at 16 DAP (0.459 vs. 0.238), indicating a shift of the mean size toward a unique value for one population with a 2-fold size difference and much larger variance. The largest protein bodies have similar sizes at 16 and 20 DAP indicating that this class of protein bodies has reached its final size. They correspond to a selective accumulation of vicilin, suggesting that legumin accumulation which occurs later probably forms separate protein bodies. The size heterogeneity observed in the small size populations reflects the active process of storage protein accumulation at this stage. It may be explained by the kinetics of protein accumulation.

### Ultrastructural analysis

At mid maturation, the ultrastructure of embryo cells showed the presence of the dense bodies, 1.5 to 3.5 μm diameter, already detected by light microscopy, and revealed the presence of an heterogeneous population of vesicles ranging from 0.2-0.5 μm diameter. The dense vesicles (0.1-0.3 μm) were filled with electron-dense aggregating material, and the multivesicular bodies (0.4-0.5 μm) displayed a set of small vesicle-like structures (Figure 4A-C). In the vacuolar compartment, the dense spherical bodies were probably integrated by autophagy. Some of them were associated with remnants of rough endoplasmic reticulum, they were surrounded by a ribosome-like layer (Figure 4 D-G). The fusion of two protein bodies was mediated by a multivesicular cluster (Figure 4 D, E). The largest smooth bodies (3 to 4 μm diameter) were observed outside the vacuolar compartement (Figure 4I), they correspond probably to final storage components.

## Discussion

The regulation of seed maturation and storage accumulation is a complex process involving the interaction of transcriptional regulation and hormone-dependent physiological controls [14,15]. The biogenesis of vicilin bodies and their accumulation kinetics were investigated in early and mid-maturation *M. truncatula* embryos, by microscopic and statistical analyses. The pattern of vicilin (7 S globulin) accumulation, one of the most abundant storage proteins in developing dicotyledonous seeds, showed evidence for two possible sorting pathways which may function when extensive storage protein accumulation occurs.

### Sub-cellular determinants of storage protein accumulation

During early *M. truncatula* seed maturation*,* the central vacuole of embryo cells has initiated a progressive fragmentation, which is concomitant with the accumulation of the first protein bodies. In pea, numerous small vacuoles per cell were also observed at early seed filling [16]. This modification of the vacuolar compartment was revealed to be an indicator for the initiation of protein accumulation. The persistence of the vegetative central vacuole in embryos, growing in vitro, with nutritional stress, was associated with the accumulation of very few protein bodies (data not shown). The presence of numerous large plastids and starch granules contribute to the production of the energy required during storage protein accumulation [17]. The two major seed storage proteins, vicilin (7 S) and legumin (11 S), are not synchronously deposited in *M. truncatula*. The early-mid maturation stage, 16–20 DAP, corresponds to vicilin accumulation, the relative abundance of legumin K transcripts and polypeptides at this stage was very low [13]. Our ultrastructural data identified dense core aggregates which correspond to the immuno-detected “vicilin-containing protein bodies” in light microscopy. Cytoplasmic organelles, such as dense vesicles and multivesicular bodies, involved in protein sorting to the storage vacuole in many plants were also observed*.*

### Kinetic of protein body formation

The large multigene vicilin family is organized in clusters on different chromosomes [18] In *M. truncatula*, the peak of vicilin gene expression occurred at 20 DAP, concomitant with a high size variability in the small protein body population. Some of this variability may be due to the asynchrony in development of embryo cells where mitotic division figures were still detected during the storage filling processes.

The distribution analysis of the protein body number and size allowed us to follow their kinetic formation. Different populations of vicilin bodies could be distinguished at each developmental stage. The three populations of small protein bodies suggested a rapid and intense storage protein accumulation process. The large protein bodies produced by fusion of the small ones have similar sizes at 16 and 20 DAP with a higher representation at 20 DAP (29.3%) compared to 16 DAP (10.8%). Those protein bodies, correspond to a selective accumulation of mature vicilin polypeptides, which were also detected in proteomic analyses at the mid-maturation embryo stage [13].

The relative abundance of the vicilin bodies at early maturation (16 DAP) raised up questions concerning vicilin accumulation which may implicate more than one pathway for the formation of protein bodies. Different pathways have been recognized for the trafficking of storage proteins. Both endomembrane progression through the Golgi, and direct endoplasmic reticulum vacuole trafficking (ERvt) pathways may function depending on the conditions [19].

Protein aggregation plays a central role in delivery to the protein storage vacuole (PSV) as suggested by Vitale and Raikel [20]. In *M. truncatula,* the vicilin aggregates correspond to what would be expected for a prevacuolar compartment (PVC). They fused via the addition of multivesicular bodies (MVB), which probably contained Golgi-derived membrane proteins. The tonoplast intrinsic protein α-TIP gene, a marker of PSV, revealed an over expression at mid-maturation [21,22], which is coherent with the presence of complete mature protein storage organelles at this stage. The accumulation of a single protein into the vicilin bodies, allowed delivering the protein as large aggregates, without need for downstream proteins to self-aggregate in the vacuole matrix [19].

### Storage protein sorting and accumulation

The role of the endoplasmic reticulum in storage protein synthesis and transport via golgi dense vesicles (DV) to protein bodies was investigated in legume species such as pea, soybean and *Phaseolus vulgaris*[3,23,24]. The sorting of protein precursors may follow different pathways, the prevalence of each pathway depending on the plant species, the developmental stage, and the composition of the newly synthesized proteins [5,19,25]*.* Although the trafficking route in most systems is the Golgi pathway, transport by autophagy and by direct ER-to-PSV traffic were also reported in cereals, pumpkin, and beans [7,26]. The sorting of storage protein precursors synthesised on rough endoplasmic reticulum requires specific signals and receptors. The vacuolar sorting receptor gene family (VSR) and the SNARE complex genes are primordial in the regulation of the vesicular traffic and the biogenesis of storage vacuoles [1,2,27,28]. The pea BP-80 gene associated to the Golgi clathrin-coated vesicles is also member of the *Arabidopsis* AtVSR gene family. In *M. truncatula*, the VSR1 homologue gene peaks in expression at 24 DAP, which is consistent with its role in legumin accumulation [4].

The signals for protein targeting to the vacuole arise from the storage protein sequence [1]. In *M. truncatula* the newly synthesized vicilin precursors form dense core aggregates, which we observed transiently associated to the rough endoplasmic reticulum. The issue of whether vicilin transits via the golgi, before being delivered to the storage vacuole remains to be resolved. The passage via the Golgi is mainly associated with the glycosylation process, but vicilin polypeptides are glycosylated while associated with the endoplasmic reticulum. The glycosylation is not a prerequisite for their subsequent transport into the storage vacuole, as many species were shown to contain polymorphic unglycosylated vicilin subunits in mature seeds [23,29]. The concept of direct ER-vacuole trafficking process was established in monocotyledons [20,26] and reported in pumpkin and soybean seeds [5,30,31]. That it may also function for vicilin accumulation in *M. truncatula* is suggested by the ultrastructural data showing an ER-derived membrane with bound ribosomes around dense bodies (1-2 μm). The analysis of phaseolin (7 S) glycan processing also indicated that its association with membranes may start within ER [1]. The multivesicular bodies identified in *M. truncatula* may also bind to the tonoplast via the Golgi- derived membrane proteins [32]. This alternative pathway, Golgi-dependent, would concern the sorting of vacuolar enzymes e.g. protease precursors, and the aquaporin α –TIP of the PSV tonoplast [1,21,24].

## Conclusions

The model of Protein Body biogenesis was proposed using a combined histology and stastistic analysis allowing to follow their kinetic formation during the process of seed storage protein accumulation. At early and mid maturation where storage globulin are mainly vicilin in *M. truncatula,* two different distributions of the vicilin-body populations were revealed, which enabled a separation of their processing. Those results are integrative and may be useful for screening mutations of candidate genes governing vicilin content. The ultrastructural data, based on the analysis of a large number of embryo cell sections, are consistent with the direct ER-vicilin body formation pathway including the possible implication of a trans–Golgi network in tonoplast membrane receptors and precursor protease sorting. The definitive evidence must be the immunogold studies with ER –resident proteins and may be considered as a futur prospect.

The ER-derived Protein bodies are potentially protected sites for the storage of post-translationally unstable proteins. They respond to the challenge of providing a means to alter the protein composition of seeds and use them to produce foreign proteins on industrial scale [33].

## Methods

### Plant material

*Medicago truncatula* cv Jemalong A17 was grown in a growth chamber at 22/19°C day/night temperature. Immature seeds were harvested at different developmental stages corresponding to early pre-storage stage until mid maturation stage between 8, and 20 days after pollination (DAP).

### Cytological procedures

Immature seeds were fixed in 4% paraformaldehyde in 50 mM phosphate buffer pH 7.2, containing 0.1% Triton X-100. After incubation at 4°C for 24 h, samples were washed in the same phosphate buffer, dehydrated using a series graded of ethanol solutions and embedded in LR White (London Resin Company), an acrylic resin with low viscosity according to the manufacturer's instructions. For histological studies in light microscopy, serial sections of 2-3 μm made on the ultra microtome were collected on Dako treated slides and stained for 1 min with 1% (w/v) Toluidine Blue O (TBO) in 0.1% M phosphate buffer, pH 6.8 to follow the developmental stages. The metachromatic stain TBO colours the protein body structures pink. Soluble and insoluble proteins were stained specifically blue with naphthol blue –black (NBB) and Fast green. For transmission electron microscopy, sections ≤ 0.1 μm were stained with uranyl acetate and lead citrate. The observations were performed with Hitachi H7500 electron microscope.

### Immuno-localisation

Affinity-purified polyclonal antibodies raised in rabbits against *Pisum sativum* vicilin were used for the detection of vicilin, one major seed storage protein [34]; a quality-control check of the antibodies on *M. truncatula* seed extract was carried out by ELISA (L. Quillien personal communication). The secondary antibodies were goat anti-rabbit conjugated to green Alexa 488 (Fluoprobes). For better penetration of the antibodies, 200 μl of 0.5% Triton X-100 in 0.1 % M phosphate buffer were applied to each slide for 10 min, then slides were washed for 2 min. Before incubating with the primary antibodies, sections were treated with a solution of 5% BSA (Bovine Serum Albumin) in 0.1% M phosphate buffer for 30 min to reduce unspecific binding, and then rinsed for 10 min. Antibody incubation occurred in a humid chamber at 37°C for 1 h. The slides were then rinsed in the phosphate buffer containing 0.1% Tween 20 3x5 min. After incubation with the secondary antibody, the slides were washed and embedded with an antifading (Vectashield) mounting medium containing propidium iodide for counterstaining. Omission of the first antibody and application of pre-immune serum served as controls and these showed no artefactual fluorescence.

Observations were performed with a Leica DMRB Microscope, equipped with bright-field and epifluorescence optics filter i3 (excitation at 450–490 nm, arrest 515 nm) which allowed us to visualize red and green fluorescence. All the digital microphotographs were taken with a 3CCD colour video camera (Sony DXC-390), in the same conditions from different individuals with the x40 and x63 objectives.

In the cotyledon cells, the protein bodies identified at 16 and 20 DAP were analysed by the image analysis program Visilog 6.7 (Noesis Gif sur Yvette France) to determine their number and size in immature seed LRW section of 2 μm thickness. The RGB images produced were compared only when they had the same enlargement. The programme dedicated to this application was able to recognise the protein bodies and measure the total and individual size; the data are formatted in a file compatible with excel, to facilitate transfer for statistical analysis. The measurements correspond to relative values.

### Statistic analysis

A finite Gaussian mixture approach was used for characterizing protein body size distribution. In this approach, the whole protein body population was treated as a mixture of more or less distinguishable groups. Mathematically, the mixed probability density function *f* is a weighted sum of *K* component densities:

(1) $$f\left(x\right)=\sum_{k=1}^{K}P\kappa \;\Phi \left(x\text{;}\;\mu_{k}\sigma_{k}\right)$$

where $\Phi \left(x\text{;}\;\mu_{k},\sigma_{k}\right)$ denotes the density function of the normal univariate distribution with mean *μ*_*k*_ and standard deviation σ_*k*_. The *P*_*K*_ are weights assigned to distribution components, with constraints that $p_{k}>0$ and $\sum_{k=1}^{K}P\kappa =1$.

Analyses of distributions expected to be mixtures rely on finding a set of overlapping components that provide the best fit to the summary distribution (Figure 3). A complete set of parameters of a mixture consists of parameters of the individual distribution components ($\mu_{\kappa },\sigma_{\kappa }$) as well as the number (*K*) and proportion (*p*_*k*_) of the components. The 'best' model was estimated by fitting models with different parameterizations and/or numbers of components to the data by maximum likelihood, and then applying the Bayesian Information Criterion (BIC) for model selection [35]. Protein body size data was log_2_-transformed before analyses. We performed all analyses using R software [36] and the mclust package [35].

## Competing interests

There is no competing interests in relation to this manuscript.

## Authors’ contributions

MD designed supervised the study performed microscopic observations and drafted the manuscript, FD performed the statistical analysis, EB conducted a part of the experimental work, and CS designed the image application in “VISILOG software”. All the authors have contributed to the manuscript, they are in agreement with the content.
